# Protective effects of melatonin receptor agonists on endotoxin-induced uveitis in rats

**DOI:** 10.22038/IJBMS.2023.67297.14749

**Published:** 2023

**Authors:** Tugba Nurcan Yuksel, Muhammed Yayla, Duygu Kose, Zekai Halici, Erdinc Bozkurt, Toktay Toktay

**Affiliations:** 1 Department of Pharmacology, Faculty of Medicine, Tekirdag Namık Kemal University, Tekirdag, Turkey; 2 Department of Pharmacology, Faculty of Medicine, Kafkas University, Kars, Turkey; 3 Department of Pharmacology, Faculty of Medicine, Sutcu Imam University, Kahramanmaraş, Turkey; 4 Department of Pharmacology, Faculty of Medicine, Ataturk University, Erzurum, Turkey; 5 Clinical Research, Development and Design Application and Research Center, Ataturk University, Erzurum, Turkey; 6 Department of Ophthalmology, University of Health Science, Ümraniye Education and Research Hospital, Department of Ophthalmology, Istanbul, Turkey; 7 Department of Histology and Embryology, Faculty of Medicine, Kafkas University, Kars, Turkey

**Keywords:** Lipopolysaccharides, Melatonin, Oxidative stress, Rats, Uveitis

## Abstract

**Objective(s):**

Melatonin has an important role in regulating a variety of physiological functions of the body. We investigated the protective effects of Agomelatine (AGO) and Ramelteon (RAME) on Endotoxin-Induced Uveitis (EIU) in rats.

**Materials and Methods:**

70 rats were randomly divided into fourteen groups. Healthy group normal saline, (IP), Uveitis group (200 μg/kg lipopolysaccharide (LPS), SC), DEX group (200 μg/kg LPS plus 1 mg/kg dexamethasone, IP), AGO20 group received 200 μg/kg LPS plus 20 mg/kg AGO, AGO40 group received 200 μg/kg LPS plus 40 mg/kg AGO, RAME2 group received 200 μg/kg LPS plus 2 mg/kg RAME, and group RAME4 received 200 μg/kg LPS plus 4 mg/kg RAME. Each group had two subgroups: the 3rd and 24th hr. The eye tissues were collected and investigated biomicroscopically (clinical manifestations and scoring, molecularly(qRT-PCR analyses of tumor necrosis factor-α (TNF-α), vascular endothelial growth factor (VEGF), and caspase 3 and caspase 9 mRNA expression), biochemically (Superoxide dismutase activity (SOD), Glutathione (GSH), and malondialdehyde levels (MDA)) and histopathologically (staining with Harris Hematoxylin and Eosin Y).

**Results:**

Melatonin receptor agonist treatment reduced the clinical score count of ocular inflammation in the uveitic rats. TNF-α, VEGF, caspase 9, and caspase 3 levels markedly decreased in the uveitic rats. Melatonin receptor agonists significantly ameliorated fixed changes in GSH, SOD, and MDA levels. Melatonin receptor agonists also ameliorated histopathological injury in eye tissues associated with uveitis.

**Conclusion:**

Melatonin receptor agonists ameliorated the inflammatory response in EIU. These findings suggest that melatonin receptor agonists may represent a potential novel therapeutic drug for uveitis treatment.

## Introduction

Uveitis is defined as a specific type of inflammation in the uvea and can affect any part of the uveal layer (1, 2). Besides clinical cases where no cause can be determined, uveitis can result from complications of autoimmune disease, infections, and chemical and metabolic injuries associated with a variety of molecular and biochemical events (3). Endotoxin-induced uveitis (EIU) model has been widely used as an experimental model for years to investigate the protective effect of new agents and to better understand the complex pathogenesis of the disease due to its similarity to human uveitis (4). 

Melatonin receptors belonging to the subtypes melatonin (MT)-1 and MT-2 have been identified in the mammalian retina. MT-1 and MT-2 receptors are found in all layers of the neural retina and the retinal pigmented epithelium (5). Ramelteon (RAME) is the first melatonin receptor agonist approved for clinical use in humans for the treatment of sleep disorders (6). RAME can mediate anti-oxidative activity through these receptors (7). There are also studies showing that RAME reduces markers related to inflammation (8). Agomelatine (AGO) is another melatonin receptor agonist and antidepressant drug widely used clinically. AGO has a pharmacological mechanism, a non-selective agonist that affects both MT-1 and MT-2 melatonin subtypes receptors and an antagonist that affects the 5-hydroxytryptamine-2C receptor. AGO creates a protective effect in multiple disease models through anti-oxidative injury and anti-apoptosis properties (9). 

In this study, we aimed to investigate the protective effects of AGO and RAME on EIU in rats with biomicroscopic (clinical manifestations and scoring), molecular (qRT-PCR analyses of Tumor Necrosis Factor-α, vascular endothelial growth factor, and Caspase-3 and Caspase 9 mRNA expression), biochemical (Superoxide dismutase activity, Glutathione, and Malondialdehyde levels) and histopathological (staining with Harris Hematoxylin and Eosin Y) methods.

## Materials and Methods

***Animals***

In this study, 70 female Wistar rats were albinos aged 4–6 months (weight: 300–330 g), which were purchased from Ataturk University Medical and Experimental Application Center Experimental Animal Laboratory. All the animals were housed in standard plastic cages under standard conditions (temperature: 22 ± 1 °C, relative humidity: 40–80%, 12 hr light-dark cycle). During the experiment, the animals had free access to standard rat food and water (*ad libitum*). All animal procedures and experiments were performed according to national guidelines for laboratory animals’ use and care. The study was approved by the animal experiments local ethics committee of Ataturk University (Protocol no: 42190979-000-E.2000058624).

***Chemicals***

LPS (E. coli O111:B4), derived from most Gram-negative bacteria’s cell walls, was purchased from Sigma-Aldrich, USA. AGO (Valdoxan© 25 mg tab) was purchased from Servier, Turkey. RAME (Ramelda© 8 tab) was purchased from Abdi İbrahim, Turkey. Thiopental sodium was obtained from Ibrahim Ethem Ulagay AS (Istanbul, Turkey) and all other chemicals for the laboratory experiments were purchased from Sigma and Merck (Germany). Dexamethasone (DEX) was obtained from the pharmacy.

***Experimental design***

The 70 rats were randomly separated into 14 groups (n = 5 per group, Table 1).

***Endotoxin-induced uveitis (EIU) model***

The EIU model was established by a single subcutaneous (SC) injection of 200 µg/kg LPS (10). AGO (20 or 40 mg/kg) (11, 12) and RAME (2 or 4 mg/kg) (13) were administered by oral gavage 30 min before the LPS administration. Dex was administered 30 min before LPS, coadministered with LPS, and 30 min after LPS injection by IP injection at a dose of 1 mg/kg (14). The rats were anesthetized with 30 mg/kg thiopental sodium. The eye tissues were collected at two time points, 3^rd^ and 24^th ^hr after LPS injection. The maximum inflammatory response in the EIU model is achieved 24^th^ hr after the LPS injection (15, 16). Therefore, the clinical activity scores of all rats were evaluated 24^th^ hr after the LPS injection. Right eye tissues were stored in a 10% formalin solution for histopathological analysis. Left eye tissues were stored at -80 °C for biochemical and molecular analysis.

***Biomicroscopic analysis***

*Clinical manifestations scoring*

Clinical features of ocular inflammation were evaluated in both eyes 24 hr after LPS injection using a biomicroscope (slit lamp). Severity was graded from 0 to 4 by an observer blind to treatment history as described previously (17, 18). The clinical grading was defined as follows: Grade 0 was defined as no obvious inflammatory response; Grade 1 as discrete dilation of the iris and conjunctival vessels (6–15 cells in a 1 * 1-mm slit area); Grade 2 as moderate dilation of the iris and conjunctival vessels with moderate flare in the anterior chamber (16–25 cells in a 1 * 1-mm slit area); Grade 3 as intense iridal hyperemia with intense flare in the anterior chamber (26–50 cells in a 1 * 1-mm slit area); Grade 4 as the clinical signs of Grade 3 plus fibrinous exudation (presence of more than 50 cells in a 1 * 1-mm slit area) and miosis.

***Molecular analyses***

*Gene expressions analyses*

This study was carried out with *in vivo* experiments and molecular-level gene studies were performed. A real-time polymerase chain reaction (RT-PCR) was planned to evaluate TNF-α, VEGF, Caspase 3, and Caspase 9 mRNA expression levels. For this, the homogenization of eye tissues, RNA isolation, cDNA synthesis, and quantitative determination of mRNA expressions were performed. 

*RNA extraction from eye tissue*

Eye tissue samples were individually weighed and homogenized with (WiseTis homogenizer Wisd). RNA extraction was performed in QIAcube. Total RNA isolation steps were continued in accordance with the manufacturer’s recommendations by using the RNeasy Mini Kit (Qiagen). The total mRNA amount was measured by using nanodrop spectrophotometry (All Sheng) at 260 nm.

***Reversed transcriptase reaction and complementary DNA (cDNA) synthesis***

cDNA generation from total RNA was performed with a High Capacity cDNA Reverse Transcription Kit. Each reaction was made with 10 **μl RNA**, and cDNA synthesis was provided with T100 Thermal Cycler (BIO-RAD) according to temperature values. The amount of cDNA was measured by nanodrop spectrophotometry (All Sheng) and the resulting cDNA was stored at -20 °C. Total RNA (10 µl), Reverse Transcription 10X Buffer (2 µl), 25 X dNTP mix (0.8 µl), 10X RT Random Primers (2 μl), MultiScribe Reverse Transcriptase (1 µl), and diethylpyrocarbonate H2O (4.2 µl) were used for cDNA synthesis reaction. 

***Quantitative Determination of TNF-α, VEGF, Caspase 3, and Caspase 9 mRNA gene expression by Real-Time PCR***

TNF-α, VEGF, Caspase 3, and Caspase 9 mRNA expression were quantified by using the TaqMan® Gene Expression Master Mix kit with B-actin as the reference gene. Amplification and quantification processes were performed on a Corbett Rotor-Gene (Thermo Fisher Scientific) device. The following TaqMan® Gene Expression Assays for 100 ng cDNA were continued by pipetting, using 100 ng cDNA, 10 µl TaqMan Master Mix, and 1 μl assay and completed to 20 µl with RNase-free H_2_O, for 40 cycles. The cycle threshold (CT) is the cycle number at which the amount of fluorescent signal in RT-PCR experiments exceeds the minimum value (threshold value) required to be observed. Ct values ​​were automatically converted into delta delta Ct (2–∆∆Ct ) (19) and the results were statistically evaluated in the APSV 19.00 package program. 

***Biochemical analysis***

After the surgical procedures, the eye tissues of all rats were cleaned and fixed with liquid nitrogen and stored at -80 °C. Subsequently, 100 mg of all tissue samples were first perfused with 1 ml PBS and then ground in liquid nitrogen using a Tissue Lyser II (Qiagen). After the grinding process, all samples were centrifuged. Superoxide dismutase activity (SOD) (20), glutathione (GSH) (21), and malondialdehyde levels (MDA) (22) from each sample supernatant and standards were measured according to the modified methods with ELISA reader (23). GSH, SOD, and MDA levels of the eye tissues were expressed respectively as U/ml protein, U/ml/protein, and u/M/ protein. All data were presented as the mean ± standard deviation results per mg of protein. 

***Protein determination***

The protein concentrations were determined by the Lowry Method, using commercial protein standards (Sigma Aldrich, Total protein kit-TP0300-1KT-(USA)).

***Histopathological analysis***

Preparation of solutions, tissue tracking procedures, preparation of sections, and staining pretreatment have been carried out in compatibility with our previous studies (24, 25). Hematoxylin and Eosin Staining Process: following the staining pretreatment, staining was done with Harris hematoxylin paint for three minutes. For histopathological assessment, stromal lamellae separations, dilatation, edema, and polymorphonuclear leukocyte areas were evaluated for eye tissues. In the present study, different areas were examined randomly in each eye, and scoring tables were created. A minimum of five fields for each tissue slide at ×100 magnification were evaluated and assigned to determine the severity of the changes using scores on a scale where: -/+: absent or almost absent, + mild positive, ++: moderate positive, and +++: severe positive (26).

***Statistical analysis***

APSV 19.00 package program was used for statistical analysis and the results were presented as means ± standard deviation (SD). Statistical analysis of molecular results (TNF-α, VEGF, Caspase 3, and Caspase 9) and biochemical results (GSH, SOD, and MDA) were performed using one-way ANOVA and the Tukey multiple comparison test. Significant differences were detected among all groups, compared with the healthy group (+ *P*<0.05, ++ *P*<0.01, +++ *P*<0.001) and compared with the uveitis group (* *P*<0.05, ** *P*<0.01, *** *P*<0.001).

**Table 1:** Experimental groups and design to investigate effects of melatonin receptor agonists on endotoxin-induced uveitis in rats

**Group**	**Group Code**	**Applied**	**Time to end the experiment**
1	Healthy	Only IP normal saline	3 hr after LPS administration
2	Uveitis	200 μg/kg LPS	
3	Dex	200 μg/kg LPS+1 mg/kg DEX	
4	AGO20	200 μg/kg LPS+20 mg/kg AGO	
5	AGO40	200 μg/kg LPS+40 mg/kg AGO	
6	RAME2	200 μg/kg LPS+2 mg/kg RAME	
7	RAME4	200 μg/kg LPS+4 mg/kg RAME	
8	Healthy	only IP normal saline	24 hr after LPS administration
9	Uveitis	200 μg/kg LPS	
10	Dex	200 μg/kg LPS+1 mg/kg DEX	
11	AGO20	200 μg/kg LPS+20 mg/kg AGO	
12	AGO40	200 μg/kg LPS+40 mg/kg AGO	
13	RAME2	200 μg/kg LPS+2 mg/kg RAME	
14	RAME4	200 μg/kg LPS+4 mg/kg RAME	

**Figure 1:**
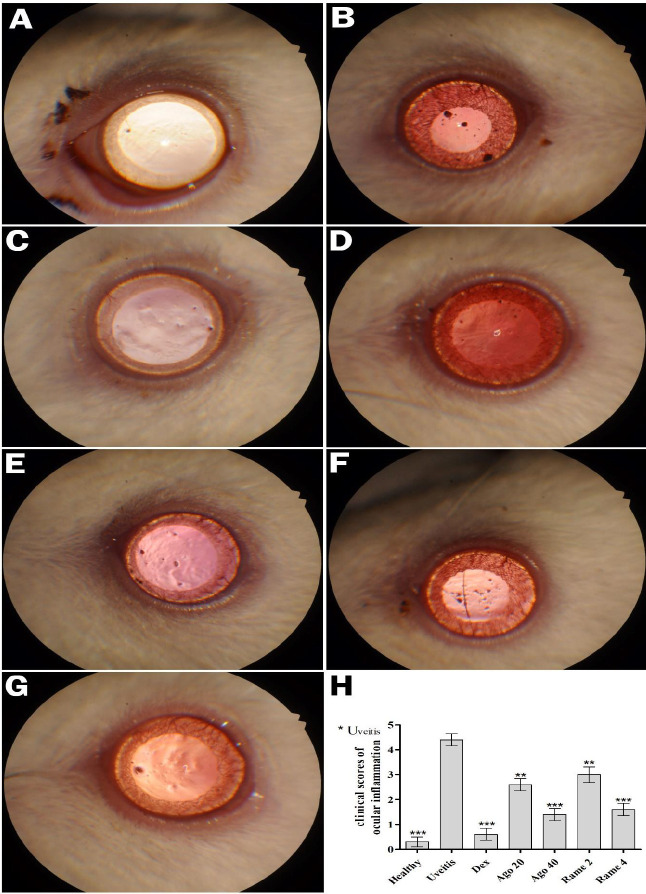
Clinical scores and biomicroscopic results of ocular inflammation 24^th ^h after LPS injection to rats

A: Healthy, B: Uveitis, C: Dex (Dexamethasone 1 mg/kg), D: AGO 20 (20 mg/kg Agomelatine), E: AGO 40 (40 mg/kg Agomelatine), F:RAME 2 (2 mg/kg Ramelteon), G:RAME 4 (4 mg/kg Ramelteon), *: Uveitis. Each bar is expressed as mean value ± SD. Significant differences were detected between all groups, compared with the uveitis group (*P<0.05, **P<0.01, ***P<0.001)

**Figure 2:**
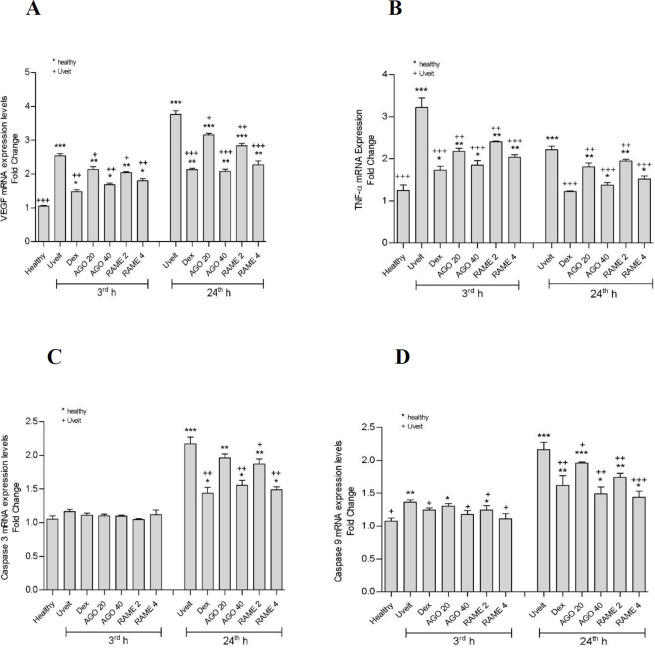
Results of molecular analyses in rat eye tissues at 3^rd^ and 24^th^ hr after LPS injection A. 3^rd^ and 24^th^ hr VEGF mRNA expression levels, B. 3^rd^ and 24^th^ hr TNF-α mRNA expression levels, C. 3^rd^ and 24^th^ hr Caspase 9 mRNA expression levels, D. 3^rd ^and 24^th^ hr Caspase 3 mRNA expression levels in the eye tissue. AGO 20: 20 mg/kg Agomelatine; AGO 40: 40 mg/kg Agomelatine; RAME 2: 2 mg/kg Ramelteon; RAME 4: 4 mg/kg Ramelteon pretreatments. The expression of mRNAs was detected using quantitative Real-Time PCR analysis. β-actin was used as the reference gene. Results are expressed as relative fold compared with healthy animals. Each bar is expressed as mean value ± SD. Significant differences were detected between all groups, compared with the healthy group (*P<0.05, **P<0.01, ***P<0.001) and compared with the uveitis group (+*P*<0.05, ++*P*<0.01, +++*P*<0.001) by one-way analysis of variance test and Tukey tests

**Figure 3:**
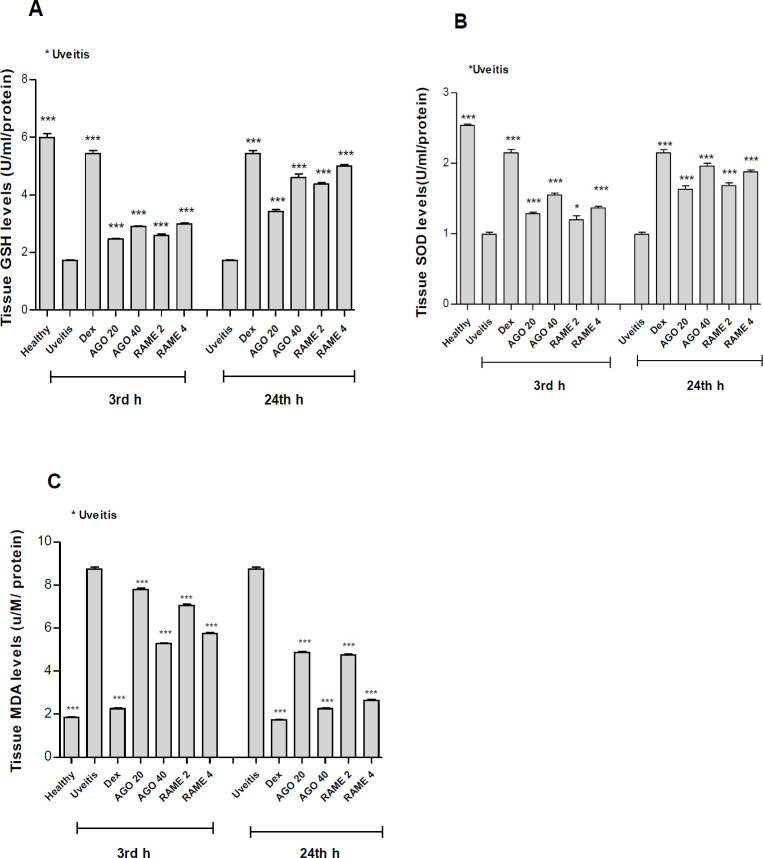
Results of biochemical analyses in rat eye tissues at the 3^rd^ and 24^ th^ hr after lipopolysaccharide (LPS) injection. A. 3^rd^ and 24^th^ hr tissue glutathione (GSH) levels, B. 3^rd^ and 24^th^ hr tissue SOD levels, C. 3^rd^ and 24^th^ hr tissue MDA levels in the eye tissue. Biochemical results of AGO and RAME pretreatments in the eye tissues. AGO 20: 20 mg/kg Agomelatine; AGO 40: 40 mg/kg Agomelatine; RAME 2: 2 mg/kg Ramelteon; RAME 4: 4 mg/kg Ramelteon pretreatments. GSH: Total glutathione levels, MDA: Malondialdehyde levels, SOD: Superoxide dismutase activities. Each bar is expressed as mean value ± SD. Significant differences were detected among all groups, compared with the uveitis group (*P<0.05, **P<0.01, ***P<0.001) by one-way analysis of variance test and Tukey tests

**Figure 4:**
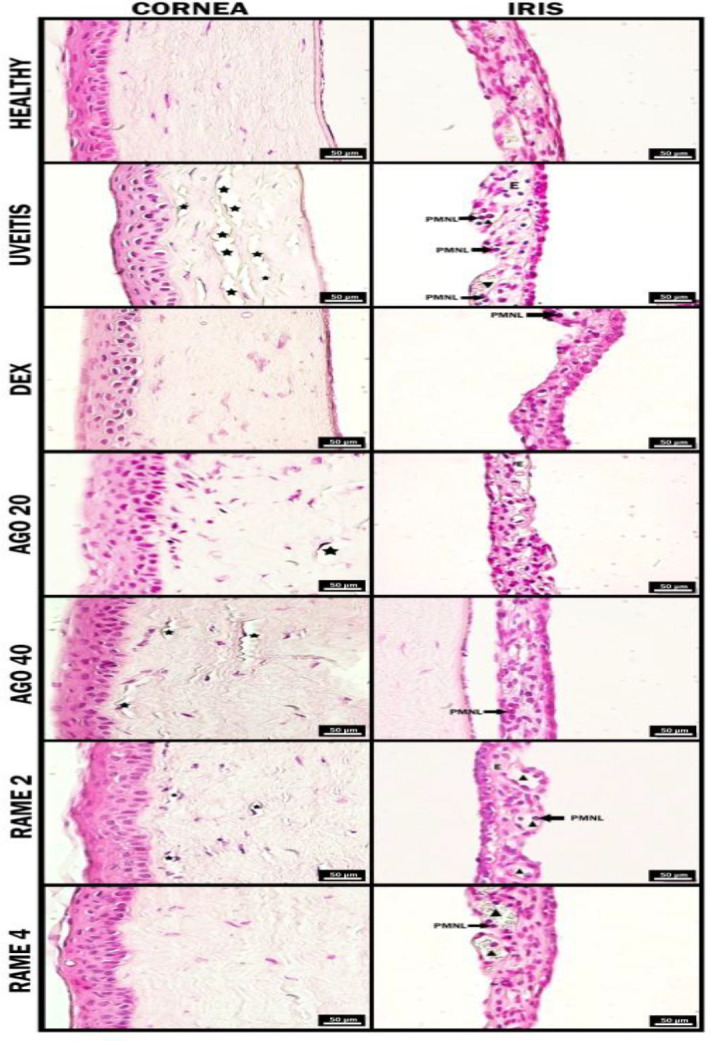
Hematoxylin-eosin stain findings of the eye tissues in 3^rd^ hr of all groups. AGO 20: 20 mg/kg Agomelatine; AGO 40: 40 mg/kg Agomelatine; RAME 2: 2 mg/kg Ramelteon; RAME 4: 4 mg/kg Ramelteon pretreatments. Star: Stromal lamellae separations, Triangle: Dilatation, E: Edema, PMNL: Polymorphonuclear leukocytes, RAME: Ramelteon

**Figure 5:**
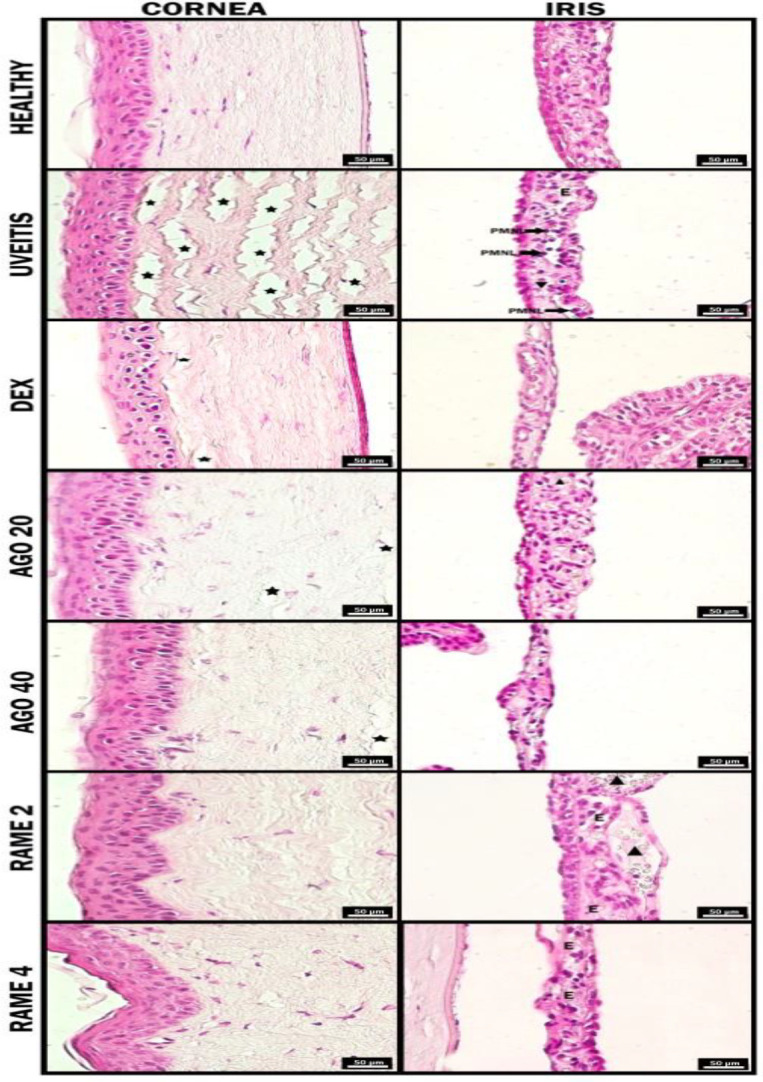
Hematoxylin-eosin stain findings of the eye tissues in 24^th^ hr of all groups. AGO 20: 20 mg/kg Agomelatine; AGO 40: 40 mg/kg Agomelatine; RAME 2: 2 mg/kg Ramelteon; RAME 4: 4 mg/kg Ramelteon pretreatments. (Star: Stromal lamellae separations, Triangle: Dilatation, E: Edema, PMNL: Polymorphonuclear leukocytes)

**Table 2:** Histopathological scores of the effect of melatonin receptor agonists on endotoxin-induced uveitis in rats

	Group code	Stromal lamellae separations	Edema and dilatation	PMNL aggregation and infiltration
3 ^rd^ h	Healthy	-	-	-
	Uveitis	+++	++	+++
	Dex	-/+	-/+	-/+
	AGO 20	-	-/+	+
	AGO 40	-/+	-/+	-/+
	RAME 2	+	+	+
	RAME 4	-	+	+
24 ^th^ h	Healthy	-	-	-
	Uveitis	+++	+	+++
	Dex	-	-	-
	AGO 20	-/+	-/+	-
	AGO 40	+	-	-
	RAME 2	-/+	++	-
	RAME 4	-/+	-	-

Dex: Dexamethasone; AGO: Agomelatine; AGO20: 20mg/kg Agomelatine; AGO40: 40mg/kg Agomelatine; PMNL: Polymorphonuclear leukocytes; RAME: Ramelteon; RAME2: 2mg/kg Ramelteon; RAME4: 4mg/kg Ramelteon pretreatments. - : Absent; -/+: Absent or almost absent, + : Mild positive, ++ : Moderate positive, +++ : Severe positive

## Results

***Biomicroscopic results***

The clinical features and clinical grading of the biomicroscopic examination of the effect of melatonin receptor agonists on EIU were analyzed in rats 24 hr after LPS injection (Figure 1). 

No inflammation signs were found in the eye tissues of the healthy group. No cellular reaction was observed in the anterior chamber (Grade 0) and the conjunctiva was not hyperemic (Figure 1-A, P<0.001). Grade 4 fibrillary reaction and intense inflammatory cellular reaction were observed in the anterior chamber, intense ciliary ejection and hyperemia were observed in the conjunctiva, miosis was observed in the pupillary area, in the eye tissues of the uveitis group (Figure 1-B, P<0.001). No cellular reaction was observed in the anterior chamber in the eye tissues of the DEX group (Grade 0), and the inflammatory reaction appeared as suppressed, similar to healthy tissue (Figure 1-C, P<0.001). A moderate cellular reaction was observed in the anterior chamber (Grade 2-3), moderate hyperemia was observed in the conjunctiva and mild miosis was observed in the pupillary area in the eye tissues of the AGO 20 group (Figure 1-D, P<0.01). A mild cellular reaction was observed in the anterior chamber (Grade 1), mild hyperemia was observed in the conjunctiva in the eye tissues of the AGO 40 group, and miosis was not observed in the pupillary area (Figure 1-E, P<0.001). A moderate cellular reaction was observed in the anterior chamber (Grade 3), moderate hyperemia was observed in the conjunctiva and miosis was observed in the pupillary area in the eye tissues of the RAME 2 group (Figure 1-F, P<0.01). Grade 2 cellular reaction was observed in the anterior chamber, moderate hyperemia was observed in the conjunctiva and miosis was observed in the pupillary area in the eye tissues of the RAME 4 group (Figure 1-G, P<0.001). Also, both AGO and RAME treatments caused a significant improvement in clinical scores of ocular inflammation compared with the uveitis group (Figure 1-H). The inflammatory response in all treated groups was significantly less than in the untreated group. Also, the most amazing improvement in the uveal tissues was observed at a dose of 40 mg/kg AGO and at a dose of 4 mg/kg RAME administration groups.

***Molecular results***

Looking at the TNF-α and VEGF mRNA expressions in the eye tissues of the uveitis group, a statistically significant increase was observed compared with the healthy group (P<0.001) (Figure 2A-B). In the AGO and RAME-administered groups, there was a statistically significant improvement compared with the uveitis group. This improvement in TNF-α and VEGF mRNA expressions increased dose-dependently at AGO 20 and 40 and RAME 2 and 4 doses. Looking at the Caspase 9 and Caspase 3 mRNA expression in the eye tissues of the uveitis group, a statistically remarkable difference raised compared with the healthy group (P<0.001) (Figure 2C-D). In the AGO and RAME-administered groups, there was a statistically significant improvement compared with the uveitis group. This improvement in Caspase 9 and Caspase 3 mRNA expressions increased dose-dependently at AGO 20 and 40 and RAME 2 and 4 doses.

The most amazing improvement in the inflammatory, apoptotic, and angiogenic parameters was acquired with the administration of AGO at a dose of 40 mg/kg and RAME at a dose of 4 mg/kg.

***Biochemical results***

It was shown that GSH level and SOD activity significantly decreased due to oxidative damage in the eye tissues of the uveitis group. In the AGO and RAME-administered groups, SOD activity, and GSH levels remarkably increased in a dose-dependent manner compared with the uveitis group (P<0.001) (Figure 3 A-B). MDA level was raised due to oxidative damage in the eye tissues of the uveitis group. In the AGO and RAME-administered groups, MDA levels markedly reduced in a dose-dependent manner compared with the uveitis group (P<0.001) (Figure 3C).

***Histopathologic findings***

The histopathologic features of the effects of melatonin receptors in the pretreatment of uveitis were shown in Figures 4 and 5. Also, the histopathological scores of the effect of melatonin receptors on the pretreatment of uveitis were shown in Table 2.

No pathological signs were found in the eye tissues of the healthy group, in the 3^rd ^and 24^th^ hr (Figures 4 and 5). In the 3^rd ^hr of uveitis groups, in the iris, remarkable vessel dilatations in the connective tissue, PMNL aggregations in the vessel, and lymphocyte and PMNL infiltrations in the connective tissue in some areas were observed. In addition, edema areas were observed in the connective tissue. On the other hand, when the cornea was examined, advanced separations were observed in the collagen fibril lamellar structures in the stroma layer (Figure 4). In the 3^rd^ hr of DEX groups, mild vascular dilatations and PMNL aggregation are seen in the iris, there was no edema in the connective tissue. Separations were rarely seen among the collagen fibril lamellae in the corneal stroma. The histopathological appearance of this group was similar to that of the relatively healthy groups (Figure 4). In the AGO 20 group, in the 3^rd^ hr, while vascular dilatations were not observed in the iris, mild edema was observed in the connective tissue. In addition, collagen fibril lamellae in the stroma of the cornea had a regular, ordered, and tightly packed appearance, and separations were rarely encountered (Figure 4). In the AGO 40 group, in the 3^rd^ hr, while vascular dilatation, edema, and inflammatory cells were not observed in the iris, PMNL aggregation was observed inside the connective tissue at a mild level. Mild separations were observed among collagen fibril lamellae in the corneal stroma (Figure 4). In the RAME 2 group, in the 3^rd^ hr, mild vascular dilatation and intravascular PMNL aggregation were observed in the iris. In the corneal stroma, separations were rarely seen among collagen fibril lamellae (Figure 4). In the RAME 4 group, in the 3^rd^ hr, mild vascular dilatation and intravascular PMNL aggregation were observed in the iris. In the corneal stroma, there were no decouples among the collogenated fibril lamellae (Figure 4).

In the 24^th^ hr of uveitis groups, vessel dilatations, PMNL aggregations, and edema areas were observed in the iris. Excessive decoupling among collogenous fibrillated lamellae was observed in the corneal stroma (Figure 4). In the 24^th^ hr of DEX groups, there were no vascular dilatations or PMNL aggregations in the iris. There were no decouples among the collogenated fibril lamellae in the stroma of the cornea. The histopathological appearance of this group was similar to the DEX 3^rd^ hr and healthy groups (Figure 5). In the AGO 20 group, in the 24^th^ hr, while vascular dilatations were not observed in the iris, collagen fibril lamellae in the cornea stroma were observed in a regular row and tightly packed appearance (Figure 5). In the AGO 40 group, in the 24^th^ hr, vessel dilatations and intravascular PMNL aggregation were not observed in the iris. In the corneal stroma, there were no separations among the collagen fibril lamellae. The histopathological appearance of this group was similar to the healthy groups (Figure 5). In the RAME 2 group, in the 24^th^ hr, while vascular dilatation was observed, inflammatory cells were not found in the iris. Separations were almost never seen among the collagen fibril lamellae in the corneal stroma (Figure5). In the RAME 4 group, in the 24^th^ hr, vessel dilatations and intravascular PMNL aggregation were not observed in the iris, In the corneal stroma, there were no separations among the collagen fibril lamellae. The histopathological appearance of this group was similar to the healthy groups (Figure 5). 

## Discussion

Uveitis induces many significant ocular comorbidities such as cataracts, cystoid macular edema, glaucomatous optic neuropathy, retinal detachment, and permanent vision loss if not diagnosed and treated promptly owing to uveitis having a complex and not exactly explained pathogenesis and multiple disease recurrence with prolonged or repeated drug treatments (27, 28). Fanlo *et al*. reported that 26.5% of the patients developed moderate vision loss (29). Therefore, it is important to follow up on uveitis cases and prevent associated complications.

The first-ever study on uveitis and melatonin was done by Toitou *et al*., and they reported that patients with a functional alteration of the retina in relation to posterior uveitis present a substantial decline of plasma melatonin levels at night. (30) Also, Abe *et al*. measured serum levels of the pineal hormone melatonin to investigate the possibility of pineal dysfunction in both rats with experimental autoimmune uveitis/pinealitis and uveitis patients in their study and reported that decreased nocturnal serum melatonin levels might be related to the presence of retinal uveitogenic antigens in uveitis patients (31). In light of this information, the effects of AGO and RAME, which are melatonin receptor agonists in the LPS-induced uveitis model were evaluated biomicroscopically, molecularly, biochemically, and histopathologically.

Primarily, we evaluated the clinical effects of AGO and RAME in the treatment of uveitis by examining properties of ocular inflammation in eye tissues 24 hr after LPS injection using a biomicroscope. Uveitis was reported to cause a serious biomicroscopic change in the eye tissue (32). Compatible with previous studies, in the present study, the uveitis group had clinical features of ocular inflammation biomicroscopically. AGO and RAME improved clinical signs caused by uveitis. 

Experimental studies with the EIU model show that cytokines have an important role in the occurrence of uveitis (33). The serious increase in cytokines and chemokine levels due to inflammation of the uvea causes changes in the activation of intracellular signal cascades in ocular tissues and the expression pattern of various inflammatory genes (34). That’s why it can be considered that changes in all these markers may be of great importance in inflammatory eye disease. In addition, it can be thought that these regulatory mechanisms may have an important role in the follow-up of the disease. The Janus kinase-signal transducer and activator of transcription (JAK-STAT) pathway mediates intracellular signals of cytokines, growth factors, and hormones, and the agents inhibiting this pathway could be effective in treating noninfectious inflammatory diseases (35). In the eye during experimental uveitis, TNF-α collects leukocytes, increases leukocyte adhesion to the vascular endothelium, activates macrophages and strains T cells, and promotes apoptosis of both established cells and infiltrating cells and previous studies have shown that TNF-α affects the JAK-STAT pathway in cells (36).  In addition, studies are showing that melatonin receptors have antibacterial properties (37, 38). Because, activation of melatonin receptors inhibits JAK2/STAT3 pathway (39), leading to cytokine and chemokine production (40). A study reported that TNF-α levels increase due to uveitis (41). Another study reported that activation of melatonin receptors attenuates the levels of pro-inflammatory cytokines such as TNF-α (42). Consistent with previous studies, we found that AGO and RAME reduced TNF-α levels induced by uveitis.

VEGF is the most potent inducer of endothelial activation and angiogenesis. It is mainly expressed in retinal neurons and glial cells and is present in only scant amounts in blood vessels (43). Under ischemic or hypoxic conditions, which occur in many neovascular diseases, retinal expression and production of VEGF dramatically increase degeneration (44). Simsek *et al*. reported that they studied patients with Behçet’s uveitis and Fuchs’ uveitis syndrome showing that VEGF levels increase due to uveitis (45). Doganlar *et al*. reported that in the *in vitro* study melatonin prevents disruption of the blood-retinal barrier and mitochondrial dysfunction by reducing VEGF receptor gene expression. It also prevents the apoptosis of retinal pigmented epithelial cells (46). Doganlar ZB *et al*. reported that melatonin can modulate genes TNF-α and VEGF, thus acting as a direct antiangiogenic molecule (47). Compatible with previous studies, we found that AGO and RAME decreased VEGF levels induced by uveitis. 

In apoptotic cascades, activation of Caspase 9 increases Caspase 3, which further induces proteolytic degradation of various cellular targets and activation of endonucleases, ultimately leading to cell death (48). In the present study, Caspase 9 and Caspase 3 levels increased in the uveitis group. AGO and RAME decreased Caspase 9 and Caspase 3 levels stimulated by uveitis. These results are compatible with those of previous studies. which also defined uveitis raises in apoptotic cascades. A study investigated the role of apoptosis in experimental autoimmune anterior uveitis solution and reported that Caspase 9 and Caspase 3 levels increase due to uveitis (49). A study reported that MT-1 receptor activation against ocular surface damage caused by hyperosmolarity improved by reducing Caspase 3 regulation (50). Zhai *et al*. in their study reported that melatonin regulates apoptosis by reducing Caspase 3 levels (51). Researchers showed that melatonin has a protective role in apoptosis by improving Caspase 3 and Caspase 9 levels (52). Consistent with the previous studies, our results suggest that AGO and RAME regulated the raise in apoptotic cascades owing to uveitis.

The present results also showed GSH level and SOD activity decreased and MDA level raised in the uveitis group. Oxidative stress plays a causal role in the complications of uveitis (53). Moreover, there are studies in the literature showing that oxidative stress, one of the markers that play an important role in the pathogenesis of uveitis, increases in the EIU model (54). Yang *et al*. reported that melatonin receptors play a protective role against oxidative stress in retinal-pigmented epithelium cells (55). Research showed that increased expression of melatonin receptors may be involved in the defense mechanisms against oxidative stress in corneal fibroblasts. In addition to direct free radical scavenging, melatonin also stimulates the activity of some anti-oxidant enzymes including superoxide dismutase (SOD), glutathione peroxidase (GPx), and catalase (CAT) which are mediated through its specific receptors including membrane receptors (56). Researchers reported that melatonin regulates oxidative stress by increasing GSH levels and SOD activity (57). Moniruzzaman *et al.* in their study reported that melatonin regulates oxidative stress by increasing GSH and SOD levels and decreasing MDA levels (58). Compatible with the previous studies, our results suggest that AGO and RAME fixed changes in the oxidant and anti-oxidant parameters including GSH, SOD, and MDA due to uveitis, indicating the strong anti-oxidant feature of melatonin receptor agonists. 

Uveitis was reported to cause a grave histological change in the eye tissue (59). Earlier studies indicated that uveitis created histopathological injury in the eye tissue including the ciliary body, retina (60), cornea, and iris (61). In the present study, the eye tissues of rats after LPS and treatment with AGO and RAME were investigated histopathologically to evaluate the potential medicinal importance of AGO and RAME in uveitis. Widespread histopathological alterations including common inflammatory cell infiltration were observed in eye tissues of the uveitis group. AGO and RAME corrected histopathologic injury in the eye tissues due to uveitis. Our histopathological findings agree with the biomicroscopic, molecular, and biochemical findings as well as the findings of earlier investigations.

## Conclusion

Based on the biomicroscopic, molecular, biochemical, and histopathological findings, we have shown that AGO and RAME significantly improved endotoxin-induced uveitis in rats. Melatonin receptor agonists can be a promising agent by contributing to alternative preventive treatment methods for uveitis with their anti-inflammatory, anti-oxidant, and antiapoptotic effects. 

## Authors’ Contributions

TNY, MY, and DK designed the experiments; TNY, EB, and ET performed experiments and collected data; TNY, MY, DK, ZH, EB, and ET discussed the results and strategy; TNY, DK, and ZH supervised, directed, and managed the study; TNY, MY, DK, ZH, EB, and ET approved the final version to be published.

## Conflicts of Interest

The authors declared that there are no conflicts of interest.
